# Determinants of viral load rebound on HIV/AIDS patients receiving antiretroviral therapy: results from South Africa

**DOI:** 10.1186/s12976-018-0082-0

**Published:** 2018-07-16

**Authors:** Claris Shoko, Delson Chikobvu

**Affiliations:** Department of Mathematical Statistics and Actuarial Sciences, University of the Free State, Box 339, Bloemfontein, 9300 South Africa

**Keywords:** HIV progression, Viral load, Lactic acidosis, Peripheral neuropathy, Non-adherence, Triple therapy

## Abstract

**Background:**

Antiretroviral therapy (ART) has become the standard of care for patients with HIV infection in South Africa and has led to the reduction in AIDS related morbidity and mortality. In developing countries, the nucleosides reverse transcriptase inhibitors (NRTIs) class are widely used because of their low production costs. However patients treated with NRTIs develop varying degree of toxicity after long-term therapy. For this study patients are administered with a triple therapy of two NRTIs and one non-nucleoside reverse transcriptase inhibitor (NNRTI).

**Method:**

In this study the progression of HIV in vivo is divided into some viral load states and a continuous time-homogeneous model is fitted to assess the effects of covariates namely gender, age, CD4 baseline, viral load baseline, lactic acidosis, peripheral neuropathy, non-adherence and resistance to treatment on transition intensities between the states. Effects of different drug combinations on transition intensities are also assessed.

**Results:**

The results show no gender differences on transition intensities. The likelihood ratio test shows that the continuous time Markov model for the effects of the covariates including combination give a significantly better fit to the observed data. From almost all states, rates of viral suppression were higher than rates of viral rebound except for patients in state 2 (viral load between 50 and 10,000 copies/mL) where rates of viral rebound to state 3 (viral load between 10,000 and 100,000 copies/mL) were higher than rates of viral suppression to undetectable levels. For this transition, confidence intervals were very small. This was quite notable for patients who were administered with AZT-3TC-LPV/r and FTC-TDF-EFV. Although patients on d4T-3TC-EFV also had higher rates of viral rebound from state 2 than suppression, the difference was not significant.

**Conclusion:**

From these findings, we can conclude that administering of any HIV drug regimen is better when based on the viral load level of an HIV+ patient. Before initiation of treatment, patients should be well equipped on how antiretroviral drugs operate including possibilities of toxicity in order to reduce chances of non-adherence to treatment. There should also be a good relationship between patient and health-care-giver to ensure proper adherence to treatment. Uptake of therapy by young patients should be closely monitored by adopting pill counting every time they come for review.

## Background

The first acquired immunodeficiency syndrome (AIDS) case emerged in the early 1980s and since then, the AIDS prevalence has been increasing [1]. Antiretroviral therapy (ART) has become the standard of care for patients with HIV infection and has led to the reduction in AIDS related morbidity and mortality [2, 3].

In South Africa, the antiretroviral therapy available at the present moment are the nucleotides reverse transcriptase inhibitors (NRTIs) class which includes among others zidovudine (AZT), didanosine (ddI), lamivudine (3TC) and stavudine (d4T) [2]. Other NRTIs include abacavir (ABC), tenofovir (TDF) and Emtricitabine (FTC) [4]. NRTIs are most preferred for HIV/AIDS patients in low income countries [5] because of their low production costs [6].

However, patients treated with NRTIs develop varying degrees of myopathy or neuropathy after long-term therapy [7]. AZT causes myopathy, ddI and 3TC cause neuropathy, d4T causes neuropathy or myopathy and lactic acidosis (LA). Studies show that d4T appears to cause lactic acidosis (LA) more frequently than ddI or AZT [8, 6]. In developed countries d4T is no longer favoured as a consequence of both short-term toxicity (lactic acidosis) and long-term toxicity (lipoatrophy and neurophathy) [6]. Neuropathy is long-term in the sense that it is usually associated with late stages of HIV disease as indicated by the presence of opportunistic infections [9]. Thus, it is highly associated with low CD4 cell count and high HIV viral load.

Science literature has successfully established the efficiency of ART in controlling HIV; however its effectiveness depends particularly on the adherence of patients to ART [1]. Adherence can be defined as the extent to which a person uses a medication according to medical recommendations, inclusive of time, dosing, and consistency [10]. Non-adherence results in antiretroviral agents not being able to maintain sufficient concentration to suppress HIV replication in infected cells and to lower the plasma viral load [11]. Poor adherence also accelerates drug-resistant HIV [11, 10].

The development of drug-resistant variants that can develop in HIV/AIDS patients under ART makes it not feasible to completely eradicate the virus [12]. This results in virological rebound and eventual disease progression [12]. But, with proper adherence to treatment, ART has the potential to suppress viral replication, often below the level of detection by commercially available tests [13]. Hirschhorn and others also identified the range of possible virologic responses which among others include failure to ever see a virologic response, decline followed by rebound, ever achieved suppression and loss of suppression after it had been achieved. This justifies the importance of viral load as a marker of treatment efficacy.

Stochastic models have proved to be the best when dealing with real life situations particularly when modelling biological phenomena such as in vivo HIV dynamics. As the HIV progresses in an individual, there is random movement between states, stochastic models are very good at handling these random variables. Stochastic processes also allow modelling the effects of covariates like stage of infection, virus subtype, presents of STIs, sexual practices, condom use, religion, education, age, gender and genes on transition intensities. In particular, time-homogeneous Markov models are usually used to model the evolution in chronic diseases [14].

### Time-homogeneous Markov modelling

Consider a model consisting of *k* = 6 states belonging to the state space *S* = {1, 2, …, k = 6}. Consider the *i*^*th*^ individual being in some state at time *t*. Let *X*(*t*) denote the state occupied by a randomly chosen individual at time *t*. Assuming that the individual’s movements obey a continuous time-homogeneous Markov process, then for 0 ≤ *s* ≤ *t* the *k* × *k* transition probability matrix *P*(*s*, *t*) with entries:

$$ {p}_{ij}\left(s,t\right)=\Pr \left\{X(t)=j|X(s)=i\right\},i;j=1,\dots, k $$

Can be specified in terms of transition intensities

$$ {q}_{ij}(t)=\left\{\begin{array}{c}\underset{\Delta  t\to 0}{\lim}\frac{p_{ij}\left(t,t+\Delta  t\right)}{\Delta  t},i\ne j\\ {}-\sum \limits_{i\ne j}{q}_{ij}(t),\kern2.75em i=j\end{array}\right. $$

Here *q*_*ij*_(*t*) are the entries of the *k* × *k* transition intensity matrix *Q*(*t*). Since our model is time-homogeneous we consider *q*_*ij*_(*t*) = *q*_*ij*_ independent of time. *Q* = (*q*_*ij*_) is the transition intensity matrix. For this model, transition probabilities are stationary such that:

$$ P\left(s,s+t\right)=P\left(0,t\right)=P(t) $$

For each of the individuals, covariates are measured. Interest centres on the relationship between the covariates and the transition intensities *q*_*ij*_ in the Markov model. Variables associated with the transition intensities are assumed to have a multiplicative effect of the form;

1 $$ {q}_{ij h}={q}_{ij}^{(0)}\exp \left({\beta}_{ij s}^T{\boldsymbol{Z}}_s\right) $$

Where ***Z*** is the *s-*dimensional vector of covariates*. β*_*ijs*_ is the vector of *s* regression parameters relating to the instantaneous rate of transition from state *i* to state *j*. ${q}_{ij}^{(0)}$ is the baseline transition intensity relating to the transition from state *i* to state *j*.

Eq. (1) can be written as a log-linear model as shown below;

2 $$ \mathit{\log}\kern0.5em {q}_{ij}={\beta}_{ij0}+{\sum}_{s=1}^p{\beta}_{ij s}{z}_s\kern0.5em \mathrm{for}\kern0.5em i\ne j=1,2,\dots \kern0.5em \mathrm{and}\kern0.5em s=1,2,\dots, p $$

where $\exp \left({\beta}_{ij0}\right)={q}_{ij}^{(0)}$ the baseline transition rates for patients in which the covariates are not mentioned, *z*_*s*_ is a s-dimensional vector of covariates and *β*_*ijs*_ represents a vector of vector of *s* regression parameters relating the transition rates from state *i* to state *j* to the covariates ${\overline{z}}_s$.

Maximum likelihood estimates of the baseline transition intensity matrix can be obtained by maximising the likelihood function with respect to the parameter ${q}_{ij}^{(0)}$. We let *T*_*ij*_, *i*; *j* = 1, …, *k*, be the total time spent by all individuals in state *i* before making a transition to state *j*. we also let *b*_*ij*_, *i*; *j* = 1, …, *k*, be the total number of transitions from state *i* to state *j*. Then the maximum likelihood estimates of the baseline transition intensities are;

$$ {q}_{ij}^{(0)}=\frac{b_{ij}}{T_{ij}},i;j=1,\dots, k $$

${\mathrm{q}}_{\mathrm{ij}}^{(0)}$ is the baseline hazard rate without (or ignoring) the effects of the covariates. In calculating ${\mathrm{q}}_{\mathrm{ij}}^{(0)}$ all *β*_*ij*0_ are chosen to be equal to zero, which means that there are no covariates effects.

Estimates of $\widehat{\beta}$ obtained by maximising the partial likelihood function are given by;

$$ L\left(\beta \right)=\prod \limits_{h=1}^n\frac{\exp \left({\beta}_{ij}^T{z}_{sh}\right)}{\sum \limits_{l\in R\left({t}_{ij,h}\right)}\mathit{\exp}\left({\beta}_{ij}^T{z}_{sh}\right)} $$

Where *z*_*sh*_ is the *s*-dimensional covariate vector for patient *h* and *R*(*t*_*ij*, *h*_) is the risk at time *t* for making a transition from state *i* to state *j*.

In this study, we explore the effects of treatment toxicity (lactic acidosis (LA) and peripheral neuropathy (PN)), non-adherence (NA), treatment line, CD4 baseline, viral load baseline, age on the changes in the level of viral load in the plasma cells. The analysis is done using a time-homogeneous Markov model with covariates. In medical research, the state of the patient at observation time is the only thing known with certainty. The researcher may know the time interval in which a transition has occurred, but not the exact time. Thus, time-homogeneous Markov models which are interval censored can handle such data [15].

In the section that follow, methods used in analysing the data are explored. This is followed by a section 3 on results and discussions. Lastly in section 4 conclusion of the findings is done.

## Methods

### Data description

The model is applied to 320 HIV-1 infected patients on anti-retroviral therapy (ART) from a Wellness clinic in Bela Bela, South Africa, from year 2005 to year 2009. These patients were observed after 3 months of treatment uptake and every 6 months thereafter. This yielded 2259 observations. From these patients 224 were females and 96 were males. 172 patients were aged between 15 and 45 and 72 were over 45 years of age. The mean age of the patients at enrolment was 40.62 years. 267 had a viral load baseline above 10,000 copies/μL and 49 had a viral load baseline below 10,000 copies/μL. At enrolment, the mean viral load was 138,208 copies/μL with a maximum of 818,600 copies/μL. 226 patients had a CD4 baseline below 200 cells/mm^3^ and 96 had a CD4 baseline above 200 cells/mm^3^. Upon initiation of treatment a number of factors were considered. These include drug toxicity which results in lactic acidosis and peripheral neuropathy. Other variables include non-adherence to treatment therapy, treatment change, treatment line and resistance to treatment. 101 patients developed lactic acidosis, 43 developed peripheral neuropathy and 36 showed some signs of non-adherence to treatment.

For each and every visit time, blood samples were obtained for each patient and stored frozen until assayed. Plasma HIV RNA was measured using an amplicator HIV-1 monitor assay kit which has a lower limit of sensitivity of 50 copies/μL.

At *t* = 0 the regimens that were mostly administered to patients were the triple combination therapy, d4T-3TC-EFV (208 patients) and d4T-3TC-NVP (92 patients). d4T and 3TC represent Stavudine and Lamivudine respectively which fall under nucleoside reverse transcriptase inhibitors (NRTI) class. EFV and NVP stand for Efavirenz and Nevirapine respectively and are from the non-nucleoside reverse transcriptase inhibitors (NNRTI) class. Table 1 below gives a frequency distribution for the different treatment combinations from *t* = 0 to *t* = 3.5 *years*.

**Table 1:** Distribution of different treatment combination for the period t = 0 to *t* = 3.5 years

	Time in Years								
	0	0.25	0.5	1	1.5	2	2.5	3	3.5
D1	208	194	168	141	95	46	18	5	3
D2	2	4	21	51	81	96	90	60	32
D3	92	77	72	63	35	23	7	1	0
D4	3	6	6	14	35	38	45	36	31
D5	0	0	0	1	1	3	8	10	10
D6	0	0	0	0	0	3	5	7	3
D7	2	2	1	2	5	4	2	2	1

Key: **D1** = d4T-3TC-EFV, **D2** = AZT-3TC-EFV, **D3** = d4T-3TC-NVP, **D4** = AZT-3TC-NVP, **D5** = FTC-TDF-EFV, **D6** = AZT-3TC-LPV/r, **D7** = Other combinations

In patients who showed some signs of non-adherence, d4T was substituted with AZT (Zidovudine). A switch from d4T-3TC-EFV (D1) to AZT-3TC-EFV (D2) was most common rising from 10 patients in the first 6 months to 92 patients in 30 months (2 and half years). During the same period the number of patients who switched from d4T-3TC-NVP (D3) to AZT-3TC-NVP (D4) rose from 6 to 45. After 1 year of treatment uptake one patient was introduced to FTC-TDF-EFV (D5) and after three and half years, the frequency increased to 10 patients. Another combination of FTC-TDF-NVP was also introduced to 3 patients after 2 years and the number rose to 7 after 3 years. AZT-3TC-LPV/r (D6) was also administered and at *t* = 0, 2 patients were administered with this triple combination. Other treatment combinations that were administered include FTC-TDF-NVP, AZT-ddI-LPV/r, d4T-3TC-LPV/r, ddI-d4T-3TC, FTC-TDF-LPV/r. However, these were not frequently administered and hence they were treated as other (D7) combinations for analysis purpose. The table below shows the frequencies for each of the treatment combinations;

**Table:** 

Drug	D1	D2	D3	D4	D5	D6	D7	Total
Frequency	879	461	370	234	56	47	212	2259

The table shows that d4T-3TC-EFV was the most frequently used drug combination.

For each visit, viral load in the plasma was also measured. In this study, if the viral load was below 50 copies/μL it recorded it as undetectable. In this study, the progression of HIV/AIDS is defined by change in viral load level. The viral load levels are divided into 5 transient states and the sixth state being the absorbing state, death. The viral load states and other factors that are likely to determine change in viral load levels are defined in the next sub-section.

### Variable coding

For this study, variables are coded as follows:

Age = $$ \left\{\begin{array}{c}1,\kern0.75em \le 45\  years\\ {}0,\kern0.75em >45\ \mathrm{years}\end{array}\right. $$, Lactic acidosis (LA) = $$ \left\{\begin{array}{c}1,\kern0.75em Yes\\ {}0,\kern0.75em \mathrm{No}\end{array}\right. $$, Peripheral neuropathy(PN) = $$ \left\{\begin{array}{c}1, Yes\\ {}0, No\end{array}\right. $$,

Non-adherence (NA) = $$ \left\{\begin{array}{c}1, Yes\\ {}0, No\end{array}\right. $$, CD4 baseline (CD4B) = $$ \left\{\begin{array}{c}1,\le 200\ \mathrm{cells}/\mathrm{m}{\mathrm{m}}^3\\ {}0,>200\ \mathrm{cels}/\mathrm{m}{\mathrm{m}}^3\end{array}\right. $$,

Gender = $$ \left\{\begin{array}{c}1,\kern1.25em male\\ {}0, female\end{array}\right. $$, viral load baseline (VLB) = $$ \left\{\begin{array}{c}1,\kern0.5em >10\ 000\  copies/\mu L\\ {}0,\le 10\ 000\  copies/\mu L\end{array}\right. $$,

Treatment Change (TC) = $$ \left\{\begin{array}{c}1, Yes\\ {}0, No\end{array}\right. $$, Treatment line (TL) = $$ \left\{\begin{array}{c}1, TL=1\\ {}0, TL=2.\end{array}\right. $$

Viral load levels (*X*(*t*)) = $$ \left\{\begin{array}{c}\mathbf{1};\kern9em VL<50\\ {}\mathbf{2};\kern4em 50\boldsymbol{\le} VL<10\ 000\\ {}\mathbf{3};\kern1.25em 10\ 000\le VL<100\ 000\\ {}\mathbf{4};\kern0.75em 100\ 000\le VL<500\ 000\\ {}\mathbf{5};\kern6em VL\ge 500\ 000\\ {}\mathbf{6};\kern10em Dead\end{array}\right. $$, Resistance (Res)=$$ \left\{\begin{array}{c}1\  if\  yes\\ {}0\  if\  NO\end{array}\right. $$

The table below shows the frequency distribution of each viral load state at *t* = 0 (baseline), that is at treatment commencement; (Table 2).

**Table 2:** Number of HIV/AIDS patients in each viral load state from t = 0 to t = 0.5 years

	Viral load levels (*X*(*t*))					
	1	2	3	4	5	6
*t = 0 years*	4	43	134	106	32	0
*t = 0.25 years*	155	123	6	4	4	24
*t = 0.5 years*	214	48	13	2	3	11

Results from Table 2 show that at t=0 years most of the patients had a viral load above 10 000 copies/μL. During the first 0.25 years of treatment uptake the majority of the patients had achieved a suppressed viral load to undetectable levels. These results show possibility of transitions, from state *i* to state *j*, between the viral load states, *X*(*t*). In this case *X*(*t*) = {1, …, 6}. We assume that the transition rate between states for any subject is governed by some covariates identified above. The effects of the covariates; age, lactic acidosis (LA), peripheral neuron (PN), gender, CD4 baseline (CD4BL), treatment line (TL), viral load baseline (VLBL), treatment change (TC), non-adherence (NA) and resistance to treatment (Res) on transition intensities, *q*_*ij*_, is assessed. The log-likelihood linking *q*_*ij*_ with the linear effects of covariates is given by:

$$ \log {q}_{ij}={\beta}_{ij0}+{\sum}_{s=1}^p{\beta}_{ij s}{z}_s\kern0.5em for\kern0.5em i\ne j,i=1,2..,5\kern0.5em and\kern0.5em j=1,2\dots, 6\kern0.5em \mathrm{and}\kern0.5em \mathrm{s}=1,2\dots, 10 $$

As defined in Eq. (2).

Thus, the transition intensity for a patient *h* in this study is given by the model:

3 $$ {q}_{ij}={q}_{ij}^{(0)}\exp \left({\beta}_{ij}^{(Age)}{Age}_h+{\beta}_{ij}^{(LA)}{LA}_h+{\beta}_{ij}^{(PN)}{PN}_h+{\beta}_{ij}^{(Gender)}{Gender}_h+{\beta}_{ij}^{\left( CD4 BL\right)} CD4{BL}_h+{\beta}_{ij}^{(TL)}{TL}_h+{\beta}_{ij}^{(VLBL)}{VLBL}_h+{\beta}_{ij}^{(TC)}{TC}_h+{\upbeta}_{ij}^{(NA)}{NA}_h+{\beta}_{ij}^{(Res)}{Res}_h\right) $$

For this model the baseline transition intensities, ${q}_{ij}^{(0)}$, refer to a patient with age category 0 (over 45 years old), no LA, no PN, Gender = 0 (female), CD4BL = 0 (above 200 cells/mm^3^, TL = 0 (second line), VLBL = 0 (over 10,000 copies/mL), no TC, no NA and no resistance. The transition intensities, *q*_*ij*_, are presented in rates per year. *q*_*ij*_ are the elements of a 6 × 6 transition intensity matrix *Q* from a continuous time-homogeneous Markov process. As indicated in Eqs. (2 and 3) can be represented by the log-linear model;

4 $$ \mathit{\ln}\ {q}_{ij}=\mathit{\ln}\ {q}_{ij}^{(0)}+{\beta}_{ij}^{(Age)}{Age}_h+{\beta}_{ij}^{(LA)}{LA}_h+{\beta}_{ij}^{(PN)}{PN}_h+{\beta}_{ij}^{(Gender)}{Gender}_h+{\beta}_{ij}^{\left(\mathrm{C}D4 BL\right)} CD4{BL}_h+{\beta}_{ij}^{(TL)}{TL}_h+{\beta}_{ij}^{(VLBL)}{VLBL}_h+{\beta}_{ij}^{(TC)}{TC}_h+{\beta}_{ij}^{(NA)}{NA}_h+{\beta}_{ij}^{(Res)}{Res}_h\Big) $$

Here *β*_*ij*_ represents the log-linear effects of the mentioned covariate on transition intensities from state *i* = 1, 2, …, 5 to state *j* = 1, 2, …, 6 for individual *h*. Computation of the estimated baseline transition intensities is done by setting all the covariates to their mean.

## Results and discussions

**Fig. 1:**
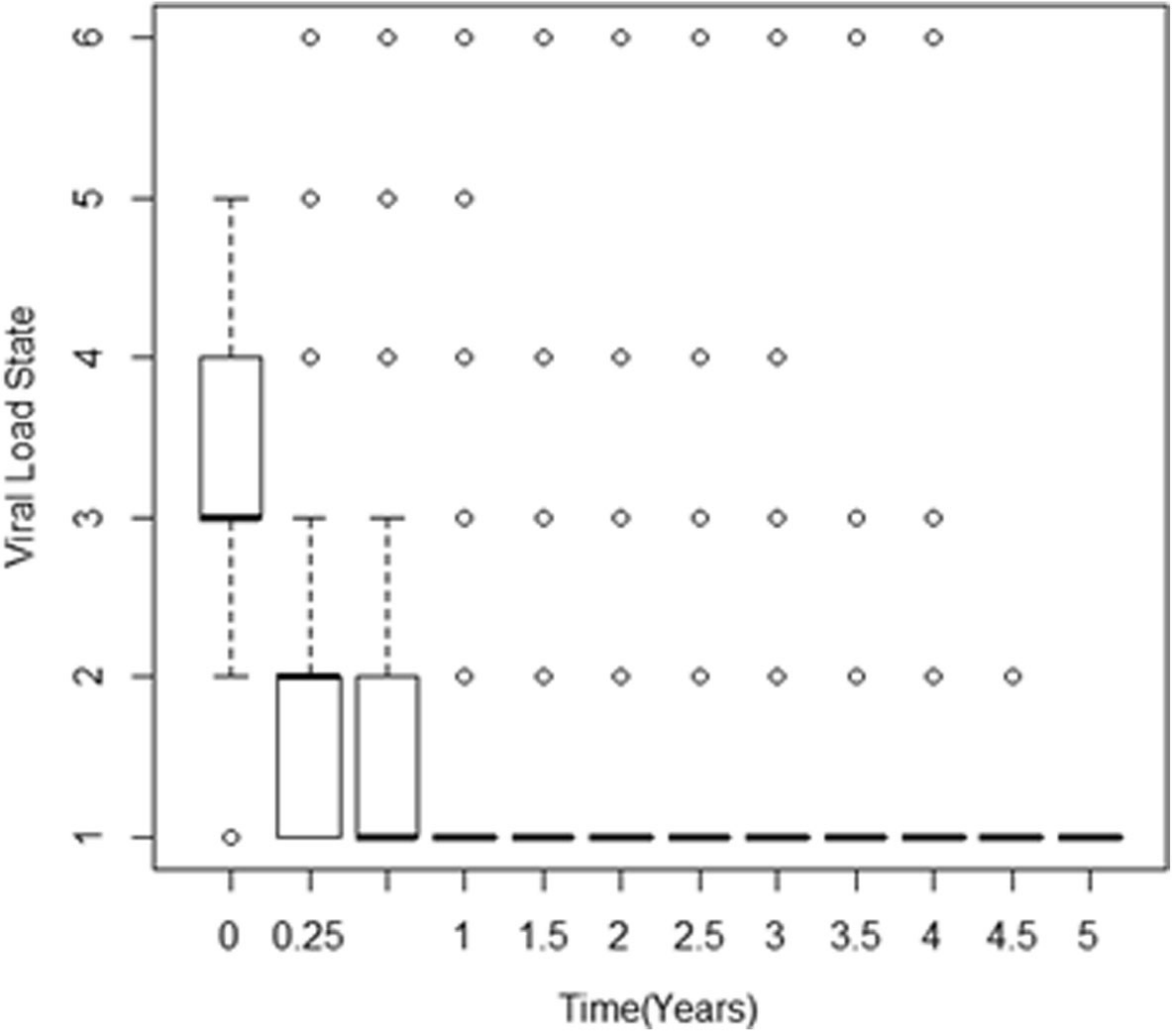
Box and whiskers diagram for the distribution of viral load levels for each visit time from initiation of therapy to 5 years. Data was collected at discrete time points, that is, at *t* = 0 years, *t* = 0.25 years, *t* = 0.5 years and after every 0.5 years thereafter

Figure 1 is a Box and whiskers plot which shows the distribution of viral load states, defined in Section 2.2, for each and every visit time which is originally considered to be discrete.

Figure 1 shows that at time equal to zero, there were no cases in state 6 since the state represents the death state. On treatment initiation, the majority of the patients were in state 3 defined by a viral load level between 10,000 and 100,000 copies/μL. After 3 months (0.25 years) the majority of the patients had moved to state 2. This is an indication of viral suppression by the antiretroviral therapy. From a period of 1 year onwards, the majority of the patients had moved to state 1, a state of undetectable viral load. However patients whose viral load is not suppressed throughout the whole period are still notable. There is need to investigate further the factors that are associated with failure of viral suppression.

### State table for transition counts

The results from Table 3 show that the highest number of deaths were recorded from state 3 which is defined by a viral load beteen 10,000 and 100,000 copies/μl. Also to note are the deaths from state 1 defined by suppressed/undetectable viral load (< 50 copies/μl), there is need to assess the determinants of deaths from this state because patients in this state have most of the virus particle cleared by the antiretroviral drug. We start off by finding estimates of the transition intensities for a continuous time Markov model without the effects of covariates. The transition intensities are estimated based on the assumption that the transition probabilities *p*_*ij*_ are known and are given as follows;

**Table 3:** Transition counts

	To					
	1	2	3	4	5	6
From 1	1109	104	16	2	1	17
2	207	98	21	4	1	8
3	80	63	22	4	0	19
4	45	54	6	2	4	9
5	6	21	0	4	2	7

$$ {q}_{ij}(t)=\left\{\begin{array}{c}\underset{\Delta  t\to 0}{\lim}\frac{p_{ij}\left(t,t+\Delta  t\right)}{\Delta  t},i\ne j\\ {}-\sum \limits_{i\ne j}{q}_{ij}(t),\kern2.75em i=j\end{array}\right.. $$

Estimates of the transition intensies are given in Table 4 below;

**Table 4:** Transition intensities from a continuous time-homogeneous Markov model

Intensities	Estimated Intensities	Confidence Interval
*q*_12_	0.4687	(0.3787,0.5801)
*q*_16_	0.01965	(0.008817,0.04379)
*q*_21_	3.446	(2.983,3.981)
*q*_23_	6.195	(2.674,14.36)
*q*_26_	0.0000386	(2.34 × 10^{−39},6.382 × 10^{29)
*q*_32_	34	(15.22,75.93)
*q*_34_	4.178	(1.551,11. 25)
*q*_36_	1.203	(0.7465,1.940)
*q*_43_	23.25	(11.88,45.52)
*q*_45_	3.651	(1.174, 11.35)
*q*_46_	0.005509	(1.84 × 10^{−45},1.64 × 10^{40}
*q*_54_	10.64	(5.962,18.97)
*q*_56_	1.466	(0.5114,4.202)
-2xLL	2799.465	

Results from Table 4 show that antiretroviral therapy plays an important role in slowing down disease progression particularly for patients in state 3 and state 4. From state 3 transitions to a better state (state 2) is more than 8 times higher than transitions to the worse state (state 4). For patients in state 4, transition to a better state is more than 6 times than transition to the worst state (state 5). However, a patient in state 2 is about twice likely to experience disease progression than recovery. This is a cause of concern since these patients have lower levels of viral load compared to patients in state 3, 4 and 5. Although transitions to better state is lower than transition to worst state for patients initially in state 2, these patients have the least transition to death compared to deaths from all the other states. This indicates that even though viral suppression is reached, HIV/AIDS patients still experience some viral rebound as supported by Hirschhorn and others in their report [12].

Results from Table 4 also show that mortality from state 4 (transition from 4 to 6) is rather too small (less than 0.05) compared to state 3 and state 5. This again is an irregularity in the fitted model which can be addressed by fitting a continuous time homogeneous Markov model with covariates effects. Thus, in the next section a continuous time Markov model with covariates is fitted.

### Effects of covariates on transition intensities

Maximum likelihood estimation of the baseline transition intensities as well as the log-linear effects for the covariates; viral load baseline (VLB), CD4 baseline (CD4B), age, gender, treatment line (TL), treatment change (TC), non-adherence (NA), lactic acidosis (LA), peripheral neuropathy (PN), resistance to treatment (Res) and triple therapy (Therapy) was done using the “msm” package in R. The log-linear model as indicated in Eq. (4) is:

$$ \mathit{\ln}\ {q}_{ij}=\mathit{\ln}\ {q}_{ij}^{(0)}+{\beta}_{ij}^{(Age)}{Age}_h+{\beta}_{ij}^{(LA)}{LA}_h+{\beta}_{ij}^{(PN)}{PN}_h+{\beta}_{ij}^{(Gender)}{Gender}_h+{\beta}_{ij}^{\left( CD4 BL\right)} CD4{BL}_h+{\beta}_{ij}^{(TL)}{TL}_h+{\beta}_{ij}^{(VLBL)}{VLBL}_h+{\beta}_{ij}^{(TC)}{TC}_h+{\beta}_{ij}^{(NA)}{NA}_h+{\beta}_{ij}^{(Res)}{Res}_h+{\beta}_{ij}^{(Therapy)}{Therapy}_h\Big) $$

where *β*_*ij*_ is the log-linear effects of the mentioned covariate on the baseline transition intensities ${q}_{ij}^{(0)}$.

On fitting the time-homogeneous model with all the covariates, we discovered that the model did not converge to a maximum likelihood. As a result confidence interval for the estimates could not be computed. The covariates effects model was fit for each of the covariates one after the other and it was discovered that treatment line (TL), gender, resistance to treatment (Res) and treatment change (TC) had no significant effects on HIV progression based on viral load. As a result, these variables were removed from the model. Results from the model with all covariates are shown in the appendices. Table 5 shows the estimated baseline transition intensities with covariates set to their mean values in the data. These represent the average intensities for the whole population of given covariates. Table 6 shows estimates of log-linear effects of each covariate on transition intensities.

**Table 5:** Baseline transition intensities

	Estimated baseline transition rates	Confidence Interval
*q*_12_	0.4938	(0.3849, 0.6335)
*q*_16_	0.0000013	(1.7 × 10^{−39}, 9.9 × 10^{26})
*q*_21_	4.008	(3.358, 4.783)
*q*_23_	49.74	(6.2 × 10^{−17}, 4.0 × 10^{19})
*q*_26_	0.000071	(9.0 × 10^{− 13}, 5617)
*q*_32_	535.5	(6.9 × 10^{− 16}, 4.1 × 10^{20})
*q*_34_	0.0090	(6.6 × 10^{−33}, 1.2 × 10^{28})
*q*_36_	0.000173	(4.3 × 10^{−23}, 6.9 × 10^{14})
*q*_43_	64.37	(0.000011, 3.8 × 10^8)
*q*_45_	0.1553	(6.1 × 10^{−21}, 3.9 × 10^{18})
*q*_46_	0.000593	(2.8 × 10^{− 103}, 1.3 × 10^{96})
*q*_54_	385.0	(0.00016, 9.5 × 10^8)
*q*_56_	0.00117	(8.7 × 10^{−18}, 1.6 × 10^{13})

**Table 6:** Log-linear effects of Covariate on Baseline Transition Intensities

	Age	VLB	CD4B	NA	PN	LA	Therapy
*β*_12_	− 0.16633 (− 0.72,0.39)	0.2244 (− 0.548, 0.997)	− 0.07828 (− 0.621,0.464)	0.04598 (− 0.721,0.813)	− 0.22434 (1.038,0.589)	− 0.3857 (− 1.01,0.238)	− 0.16391 (− 0.291,-0.036)
*β*_16_	4.90404 (− 27.45,37.25)	4.3865 (− 30.94,39.71)	4.37298 (− 29.56,38.30)	4.84416 (− 19.60,29.29)	−6.46244 (− 304.5291.5)	−5.4473 (− 132.3121.3)	− 0.62595 (− 10.66,9.409)
*β*_21_	− 0.37704 (− 0.747,-0.007)	−0.3692 (− 0.958,0.219)	0.32956 (− 0.033,0.691)	− 1.37557 (− 1.941,-0.810)	0.13742 (− 0.463,0.738)	−0.2188 (− 0.664,0.227)	−0.26823 (− 0.362,-0.1743)
*β*_23_	0.57497 (− 1.807,2.957)	6.9272 (− 9.54,23.39)	− 7.29998 (− 140.9126.3)	3.98173 (− 13.81, 21.77)	1.35016 (− 1.674,4.37)	6.0407 (− 10.04,22.11)	0.07194 (− 0.569, 0.714)
*β*_26_	−2.30421 (− 4.149,-0.459)	4.5439 (− 29.65,38.73)	6.73053 (− 26.55,40.01)	− 7.68210 (− 42.08,26.72)	−8.15962 (− 44.34,28.01)	−8.0351 (− 38.79, 22.71)	− 0.11284 (− 0.536, 0.311)
*β*_32_	0.10538 (− 2.033, 2.244)	5.5407 (− 10.91,21.99)	−7.48353 (− 141.2126.2)	2.05465 (− 15.80, 19.91)	0.90591 (− 1.60,3.41)	5.1128 (− 10.99, 21.21)	− 0.02301 (− 0.636, 0.590)
*β*_34_	6.34668 (− 22.04,34.74)	0.9591 (− 364,366)	6.36080 (− 24.03,36.75)	− 0.39441 (− 5.74,4.95)	0.75615 (− 2.91, 4.43)	− 7.5396 (− 96.93, 81.85)	0.18151 (− 0.364, 0.727)
*β*_36_	−0.56368 (− 91.13,90.0)	− 0.5756 (− 167.8166.7)	−0.32611 (− 100.58,99.9)	−1.74039 (− 129.,126)	−0.02814 (− 118.8118.7)	−0.5560 (− 101.8100.7)	0.17006 (− 22.594,22.93)
*β*_43_	0.51266 (− 1.15,2.18)	− 5.2372 (− 101.6,91.18)	0.88887 (− 0.728,2.50)	−0.60486 (− 4.507, 3.29)	−0.17207 (− 1.94,1.59)	0.4315 (−1.64,2.50)	0.05339 (− 0.403, 0.510)
*β*_45_	1.13450 (− 95.6,97.88)	1.0766 (− 182.2184.4)	−3.17689 (− 23.95,17.59)	6.33428 (− 24.76, 37.43)	−4.56027 (− 39.83, 30.71)	−1.2263 (− 28.31, 25.8)	0.12320 (− 1.479, 1.726)
*β*_46_	−0.01190 (− 87.59,87.56)	−0.7223 (− 1.412,1.41)	−0.55243 (− 89.7,88.6)	−0.98022 (− 136.4134.4)	−0.33782 (− 126.7126.1)	0.7166 (− 100.7102.2)	0.16568 (− 7.369, 7.701)
*β*_54_	−6.22114 (− 52.9,40.48)	−1.3299 (− 9.66,7.001)	2.39466 (− 2.147,6.94)	2.10654 (− 21.43, 25.6)	−0.578 (− 5.37,4.22)	2.3389 (− 7.64,12.31)	0.39170 (− 0.892, 1.676)
*β*_56_	0.00842 (− 85.69,85.71)	3.1076 (− 29.67,35.89)	− 3.87100 (− 47.67,39.93)	−2.54236 (− 69.13, 64.0)	−0.86234 (− 68.27,66.54)	− 1.6107 (− 23.59, 20.37)	−0.01408 (− 9.110,9.082)
	−2xLL =1691.177						

Table 5 above shows the baseline transition intensities for the model with covariates. Results from Table 5 now show a decreasing trend in the transition rates as the viral load becomes more and more suppressed. As a result, the undetectable viral load state (state 1) has the lowest transition rates to death. This result justifies the need to include covariates effect in Markov models. The results also show that for patients with a viral load level greater than 2, transition rates to better states are higher than transition rates to worse states. This is quite pronounced for patients initially in state 3 where transition to a better state (state 2) is 535.5 which is quite high compared to transition to the worse state (state 4) which is equal to 0.0090. However, there is a treatment challenge as patients make transitions from state 2. These patients tend to have a viral rebound resulting in transition to a worse state (state 3) being far much higher than transitions to an undetectable viral load state (state 1). This means that for HIV patients in this cohort, achieving undetectable viral load was a challenge. In the next Table are the effect age, viral load baseline (VLB), CD4 baseline (CD4B), non-adherence (NA), peripheral neuropathy (NA), Lactic acidosis (LA) and Triple therapy (Therapy). Estimates of the confidence intervals are also given. Results from Table 6 below show maximum likelihood estimates of the log-linear effects of the variables on the baseline transition intensities.

Results from Table 6 show that for patients with non-adherence (NA) to treatment there is a reduction of transitions from a viral load level of 2 (viral load between 50 and 10,000 copies/ μL) to a viral load level of 1 (undetectable viral load). For the same group of patients, there is an accelerated rate of transition from state 2 to state 3 (between 10,000 and 100,000 copies/μL). Non-adherence to treatment also cause an accelerated rebound of viral load from state 4 (between 100,000 and 500,000 copies/μL) to state 5 (viral load over 500,000 copies/μL). From all the states, the results also show an accelerated viral rebound for patients who developed some resistance to treatment compared to those who did not. This is shown by very high positive values of *β*_*ij*_’s for cases in which *j* is a worse state compared to *i*. Patients with peripheral neuropathy also have accelerated transition rates from state 2 to state 3. Although transition from state 2 to state 1 are accelerated, the rate is slower than that from state 2 to state 3. From the results it can also be noted that having lactic acidosis (LA) accelerates transition from state 2 to state 3 more than either having peripheral neuropathy or non-adherence. Patients who enrolled when their CD4 cell count was below 200 cells/mm^3^ have higher transition rates from a viral load level between 10,000 and 100,000 copies/μL (state 3) to a viral load between 100,000 and 500,000 copies/μL (state 4). Having a viral load baseline level greater than 10,000 copies/μL at enrolment increases viral rebound from state 2 to state 3.

The different treatment combinations give precise estimates of the log-linear effects as shown by the confidence intervals that are narrow. Given the different combination therapy administered to patients, transitions to viral rebound are greater than transitions to viral suppression for patients with viral load 2 and 3 (viral load between 50 and 100,000 copies/μL).

From the different combination therapy that was administered to the patients, D4T-3TC-EFV was the most frequently administered triple therapy with 889 cases, followed by AZT-3TC-EFV and D4T-3TC-NVP and AZT-3TC-NVP with 475; 431 and 279 cases respectively. Table 7 shows the transition intensities for the different drug combinations.

**Table 7:** Transition intensities for various drug combinations on viral load states

	Baseline	D1	D2	D3	D4	D5	D6	D7
*q*_12_	0.493066 (0.400,0.607)	0.607293 (0.458,0.806)	0.515483 (0.415,0.64)	0.4376 (0.353,0.543)	0.371404 (0.281,0.491)	0.3153 (0.216,0.460)	0.2676 (0.164,0.437)	0.227 (0.124,0.418)
*q*_16_	0.001107 (0.00004,30.57)	0.002452 (0.0001,371)	0.001311 (0.00006,26.32)	7.013e-04 (0.00006,821)	0.000375 (8.4e-14,1.7e + 06)	2.005e-04 (4.1e-18,9.8e + 09)	1.072e-04 (1.4e − 22,8.1e + 13)	5.735e-05 (4.1e-27,7.8e + 17)
*q*_21_	3.279872 (2.82,3.8122)	4.612591 (3.802,5.596)	3.527413 (3.027,4.11)	2.698 (2.289,3.180)	2.062904 (1.656,2.57)	1.578 (1.174,2.12)	1.206 (0.83,1.763)	0.9226 (0.578,1.472)
*q*_23_	6.644026 (1.788,24.68)	6.063371 (1.422,25.86)	6.515630 (1.774,23.92)	7.002 (1.642,29.86)	7.523862 (1.21,46.77)	8.085 (0.792,82.53)	8.688 (0.489,154.4)	9.336 (0.293,297.6)
*q*_26_	0.391710 (0.279,0.549)	0.452130 (0.259,0.79)	0.403885 (0.291,0.561)	0.3608 (0.216,0.603)	0.322291 (0.133,0.779)	0.2879 (0.0796,1.041)	0.257 (0.047,1.406)	0.2297 (0.027,1.906)
*q*_32_	38.175291 (11.11,131.2)	39.308653 (10.41,148.4)	38.414356 (11.41,129.3)	37.54 (9.342,150.8)	36.686336 (6.223,216.3)	35.85 (3.737,344.0)	35.04 (2.134,575.2)	34.24 (1.187,987.3)
*q*_34_	3.161374 (1.161,8.61)	2.509951 (0.829,7.60)	3.009489 (1.124,8.061)	3.608 (1.149,11.33)	4.326610 (0.968,19.33)	5.188 (0.745,36.14)	6.220 (0.549,70.43)	7.458 (0.397,140.2)
*q*_36_	0.010004 (0.00005,212.1)	0.008059 (0.00001,59.72)	0.009553 (0.000001,681)	0.011 (0.00019,6700)	0.013423 (0.00011,1508)	0.016 (0.00028,8987)	0.019 (0.00053,6690)	0.02236 (0.00092,5419)
*q*_43_	19.868383 (10.22,38.61)	18.564606 (10.41,33.12)	19.582706 (10.73,35.75)	20.66 (8.413,50.72)	21.789 (5.987,79.31)	22.98 (4.128,128.0)	24.24 (2.809,209.2)	25.57 (1.900,344.3)
*q*_45_	4.354376 (0.513,36.90)	3.723129 (0.893, 15.53)	4.211261 (0.679,26.127)	4.763 (0.209, 108.3)	5.387908 (0.053,545.4)	6.094 (0.0013,290.2)	6.893 (0.003,1578)	7.797 (0.0007,8679)
*q*_46_	0.00686 (0.00007,666.4)	0.005563 (0.00002,1335)	0.006565 (0.00006,710)	0.0077 (0.000019,3109)	0.009145 (0.00001,4305)	0.011 (0.00004,2594)	0.012 (0.00005,28,140)	0.01503 (0.00005,4004)
*q*_54_	17.711355 (3.557,88.2)	10.764470 (5.385,21.52)	15.925970 (4.35,58.27)	23.56 (1.960,283.2)	34.860406 (0.829,1466)	51.58 (0.345,7711)	76.31 (0.1426,4082)	112.9 (0.0588,2168)
*q*_56_	0.029928 (0.0004,2199)	0.030468 (0.00062,14,870)	0.030042 (0.00038,2347)	0.02962 (0.00004,2153)	0.029208 (0.00056,15,210)	0.02880 (0.00018,45,560)	0.02840 (0.00035,22,650)	0.02800 (0.00055,14,060)

Key: **D1** = D4T-3TC-EFV, **D2** = AZT-3TC-EFV, **D3** = D4T-3TC-NVP, **D4** = AZT-3TC-NVP, **D5** = FTC-TDF-EFV, **D6** = AZT-3TC-LPV/r, **D7** = Other combinations

Results from Table 7 shows that narrow confidence intervals for transition intensities from state 1 to 2 (rebound from an undetectable viral load to a viral load between 50 and 10,000 copies/μL), 2 to 6 (deaths from a viral load level between 50 and 10,000 copies/μL) and 2 to 1 (transition from a viral load between 50 and 10,000 copies/μL to an undetectable viral load). This indicates that the continuous time Markov model for the different drug combinations predicts better these transitions compared to all the other transitions. However, confidence intervals for the deaths from state 1, 3, 4 and 5 are very wide. This could be due to the smaller numbers of deaths for patients in this cohort. Highest rates of mortality are recorded for patients with viral load level between 50 and 10,000 copies/μL, but from all the other states mortality rates are very low.

Overall, the model shows higher transition rates to viral suppression compared to the transitions to viral rebound. For patients with a viral load between 10,000 and 100,000 copies/μL, drug combination d4T-3TC-EFV has the highest transition rates to recovery followed by the triple combination AZT-3TC-EFV, d4T-3TC-NVP, AZT-3TC-NVP, FTC-TDF-EFV, AZT-3TC-LPV/r respectively. When the viral load is still above 100,000 copies/μL, the triple combination AZT-3TC-LPV/r gives the best results followed by FTC-TDF-EFV, AZT-3TC-NVP, d4T-3TC-NVP, d4T-3TC-EFV, d4T-3TC-NVP, AZT-3TC-EFV in that order. However, for this cohort AZT-3TC-LPV/r as not frequently administered. For patients in state 2, viral rebound to state 3 is greater than viral suppression to undetectable levels and these rates of viral rebound are the highest for patients being administered with triple combinations AZT-3TC-LPV/r and FTC-TDF-EFV.

Below are the expected amount of time spent in each state from *t* = 0, the present time and death (absorbing state). For a patient in state *r* at *t* = 0, the expected total time the patient is to spend in state *s* before relapse to death is given by:

$$ {L}_s=\underset{0}{\overset{t}{\int }}{p}_{rs}(u) du $$

where *p*_*rs*_ is the probability of transition from state *r* to state *s*. The value of state r is by default set to be 1 (the undetectable viral load state). The results are given below.

**Table:** 

State 1	State 2	State 3	State 4	State 5	State 6
18.53012518	2.48700930	0.43269999	0.06880021	0.01688621	Infinity

Thus the patients are forecasted to spend approximately 18.5 years in a state of undetectable viral load (state 1) and in the other states patients are expected to spend less than 2.5 years since these are temporal states. This is evidenced by the fact that throughout the 5 year study period, only 17.8% of the patients were reported dead with 10.9 points occurring during the first 6 months.

### Assessment of the fitted model

**Fig. 2:**
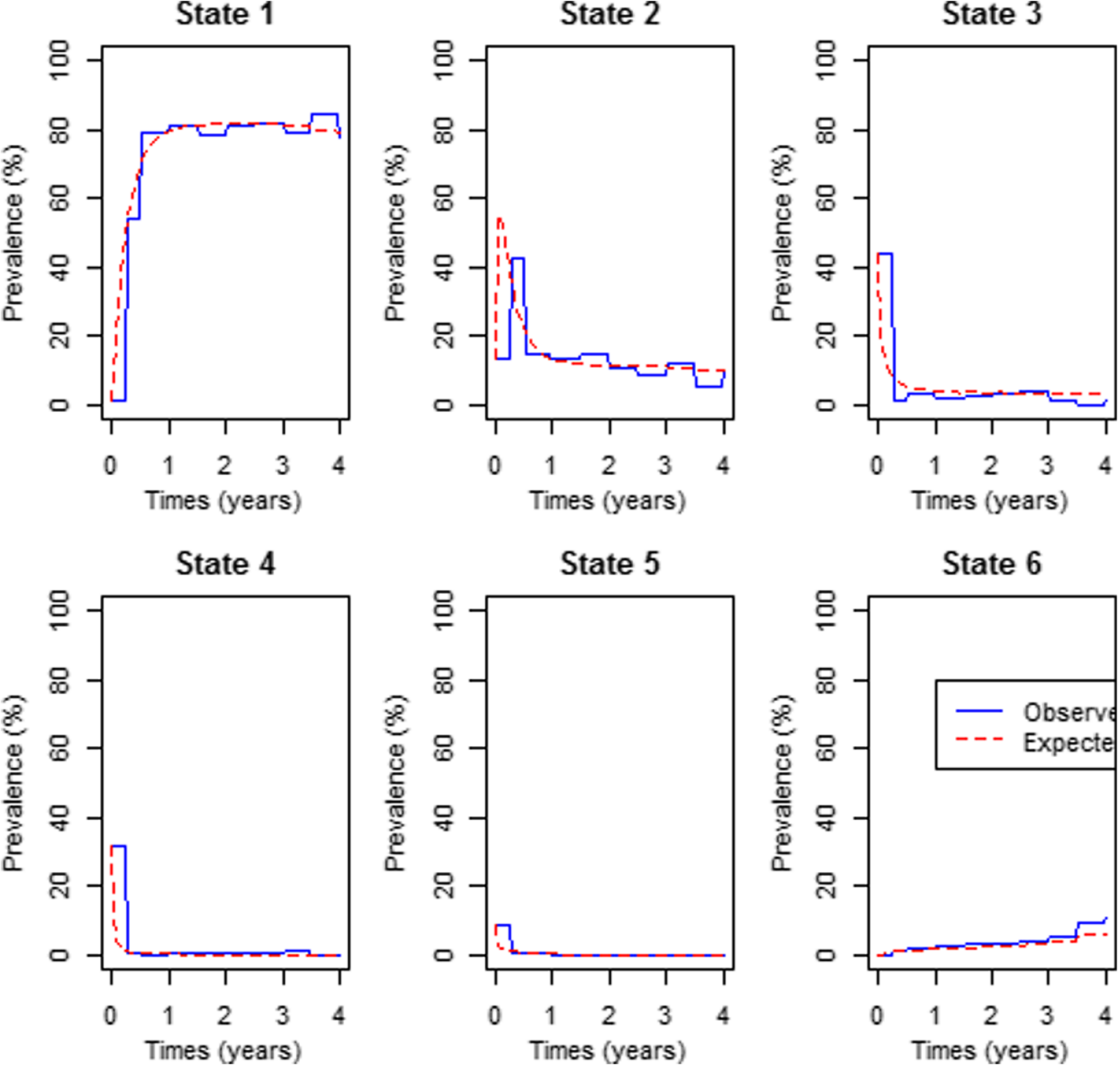
A comparison of the observed and expected percentage prevalence for the effects of Covariates on Viral Load levels. Prevalence is averaged over the covariates observed in the data, that is, viral load baseline (VLB), CD4 baseline (CD4B), age, non-adherence (NA), lactic acidosis (LA), peripheral neuropathy (PN), and triple therapy (Therapy)

**Fig. 3:**
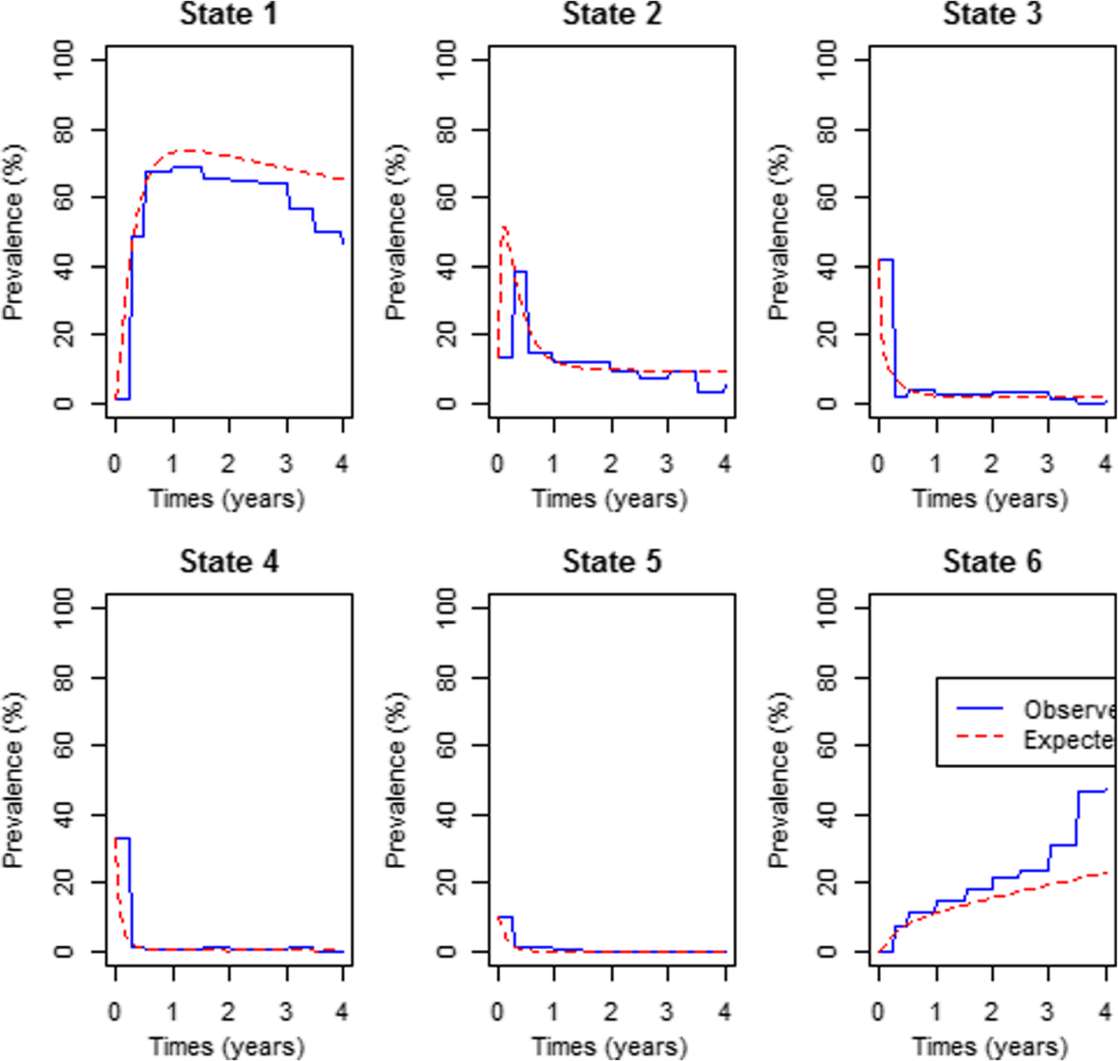
A comparison of the observed and expected percentage prevalence for the model with different combination therapy. Prevalence is averaged over the different combination therapies observed in the data, that is, D1 = D4T-3TC-EFV, D2 = AZT-3TC-EFV, D3 = D4T-3TC-NVP, D4 = AZT-3TC-NVP, D5 = FTC-TDF-EFV, D6 = AZT-3TC-LPV/r, D7 = Other combinations

In order to assess the goodness of fit of the continuous time-homogeneous Markov model for the effects of covariates, the expected percentage prevalence is plotted against the observed percentage prevalence. The prevalence is averaged over the covariates observed in the data. The percentage prevalence is plotted as functions of time for each viral load state. Figures 2 and 3 show the pravalence plots for the effects of all covariates including treatment therapy and the model for the effects of treatment therapy respectively for each state.

The results from the plots in Fig. 2 show perfect fit of the model to the observed data. In addition to that, the plots show that the percentage prevalence for state 1 increase rapidly in the first year. This shows that under normal circumstances patients on antiretroviral therapy are expected to attain an undetectable viral load in less than a year post treatment commencement. For this same state it also shows that the percentage prevalence becomes stable after a year. Although at *t =* 0 state 1 had the least percentage prevalence, from 1 year onwards about 80% of the patients had attained an undetectable viral load (state 1). States 2 and 3 had the highest percentage prevalence at *t = 0*, however in less than 6 months of treatment uptake, the percentage prevalence had dropped (Fig. 3).

Results from Fig. 3 show that if we only consider the effects of treatment therapy without considering the effects of other covariates, the fitted model underestimates death prevalence as well as state 1 prevalence. We further perform a  likelihood ratio test to compare the fitted models, that is, model without covariates (VLS3.msm), model with all covariates except combination therapy (VLS3.cov1.msm), model with all the covariates including the combination therapy (VLS3.cov11.msm) and the model for the combination therapy only (VLS3.cov.msm). The results from the likelihood ratio tests and the log-likelihoods of the preferred models are shown below.

Results from Table 8 show that the model with all covariates including the combination therapy, is the best model for this data. This model has got the maximum likelihood estimates leading to a lowest − 2*log-likelihood and also the results from the likelihood ratio test are in favour of the model with covariates including combination therapy.

**Table 8:** Likelihood ratio tests for the comparison of the fitted models and the −2 Log Likelihood (−2LL) for the preferred model

Models Tested	Preferred Model	-2 log LR	df	p	-2 LL
VLS3.msm & VLS3.cov.msm	VLS3.cov.msm	66.97594	13	2.9 × 10^−9^	2635. 207
VLS3.msm&VLS3.cov1.msm	VLS3.cov1.msm	970.1007	78	10^−4^	1732.082
VLS3.cov.msm&VLS3.cov1.msm	VLS3.cov1.msm	903.1247	65	10^−4^	1732.082
VLS3.cov1.msm&VLS3.cov11.msm	VLS3.cov11.msm	40.90497	13	9.9 × 10^−5^	1691.177

A further assessment of the fitted models is done using the Akaike Information Criteria (AIC). For each model, *AIC* =  − 2 ×  *log* (*likelihood*) + 2(*k*) where *k* is the number of parameters in the model. For example, the model with covariates excluding the combination therapy (VLS3.cov.msm) has got 26 degrees of freedom and −2 ×  *log* (*likelihood*) = 2635.207, thus AIC = 2635.207 + 2 × 26 = 2687.207 as shown in Table 9 below. The model with the smallest AIC is considered the most effective distribution of the data. The results are shown in Table 9 below.

**Table 9:** AICs for the fitted models

Model	VLS3.msm	VLS3.cov.msm	VLS3.cov1.msm	VLS3.cov11.msm
AIC	2728.183	2687.207	1914.082	1899.177

Results from Table 9 shows that the model with covariates has the smallest AIC. This confirms the results obtained from Table 8 that the time-homogeneous Markov model with covariates gives the most effective distribution of the data.

## Conclusion

This study is carried out from a cohort of HIV+ patients receiving antiretroviral therapy in Bela Bela South Africa. Using the data, four nested continuous time homogeneous Markov models were fitted. The first one had no effects of covariates, the second one had the log-linear effects of covarites without combination therapy, the third one had the log-linear effects of different combination therapy and the last one had the log-linear effects all covariates including combination therapy. These covariates include; adherence to treatment, development of drug toxicity in the form of peripheral neuropathy and lactic acidosis, change in treatment therapy, gender, age, CD4 baseline and viral load baseline and resistance to treatment on transition intensities are assessed. From the fitted model the variables; gender difference and change of treatment do not exhibit any significant effects on the transition intensities hence they were removed from the model.

The fitted models were assessed using the AICs and pairwise likelihood ratio test. The continuous time Markov model with all covariates including combination therapy had the lowest AIC an indication that it gives the best fit of the data than all the other models and also the likelihood ratio test revealed that it fits well. This as further confirmed by the likelihood ratio test which showed that the model with all covariates including combination therapy fits significantly better than any other model nested within it. Exclusion of covariates had caused some irregularities in predicting mortality which were corrected after the inclusion of covariates effects in the fitted model.

The results from the analysis showed that although close to 80% of the individuals had their viral load suppressed to undetectable levels in the first year of treatment uptake, some viral rebound were also notable particularly from state 2 (viral load level between 50 and 10,000 copies/μL) to state 3 (viral load level between 10,000 and 100,000 copies/μL). Further analysis showed that this rebound was accelerated by non-adherence to treatment, lactic acidosis and resistance to treatment. However, for patients who developed peripheral neuropathy, there is an accelerated transition to both viral rebound and viral suppression from state 2 although the rate of viral rebound is greater than the rate of viral suppression. For these patients when the viral load is above 100,000 copies/μL there are reduced rates of viral suppression. This corroborates work done by Simpson who argued that greater incidences of peripheral neuropathy are in the strata of patients with plasma HIV RNA levels greater than 10,000 copies/μL [9]. Patients who initiated treatment therapy with a viral load level above 10,000 copies/μL also had some notable viral rebound from state 2 (viral load level between 50 and 10,000 copies/μL) to state 3 (viral load level between 10,000 and 100,000 copies/μL). Considering the different combination therapy administered to patients, rates of viral rebound are greater than the rates of viral suppression especially for patients who were administered with FTC-TDF-EFV and AZT-3TC-LPV/r for patients in state 2. Highest rates of mortality are also recorded for patients with viral load level between 50 and 10,000 copies/μL, but from all the other viral load states mortality rates are very low. In particular, for patients with viral load level between 10,000 and 500,000 copies/μL, lowest transition rates were recorded especially for patients administered with d4T-3TC-EFV and AZT-3TC-EFV.

Disease progression is faster on patients below the age of 45 compared to patients over 45 years in the cohort. This shows that older patients have a better understanding of the treatment therapy resulting in a better adherence to the treatment therapy. On the other hand, young patients have substantial challenges in achieving level of adherence necessary for successful therapeutic outcomes.

From state 3 (viral load level between 10,000 and 100,000 copies/μL), rates of viral suppression are higher than the rates of viral rebound particularly for patients administered with d4T-3TC-EFV. A CD4 baseline below 300 cells/mm^3^ accelerates the transitions from state 3 to state 4 (between 100,000 and 500,000 copies/μL). This is also the case with younger patients below the age 40 years.

Non-adherence accelerates viral rebound for patients with viral load levels between 100,000 and 500,000 copies/μL (state 4). This supports the issues raised by Chesney [11] that without proper adherence antiretroviral agents are not maintained at sufficient concentration to suppress HIV replication. Hence the need to have a proper patient-health-care provider relationship and also count check of the pills (counts) by asking patients to bring the empty packs.

Overall, the model shows higher transition rates to viral suppression compared to the transitions to viral rebound.

This study has revealed the major attributes to viral rebound on HIV+ patients which is notable as patients attain a viral load level between 50 and 10,000 copies/μL. The major attributes were non-adherence, lactic acid, resistance to treatment, and different combination therapy like AZT-3TC-LPV/r and FTC-TDF-EFV. However, assuming that the patient was initially in state 1 (the undetectable viral load state) he is expected to spend approximately 18.5 years in state 1 before he dies. This is evidenced by the fact that throughout the 5 year study period only 17.8% of the patients were reported dead with 10.9 points occurring during the first 6 months.

Hence the need to administer HIV drug regimens is better based on the viral load level of a patient. Before initiation of treatment, patients should be well equipped on how antiretroviral drugs operate including possibilities of toxicity in order to reduce chances of non-adherence to treatment. There should also be a good relationship between patient and health-care-giver to ensure proper adherence to treatment. Uptake of therapy by young patients should be closely monitored by adopting pill counting every time they come for review.
